# Words Analysis of Online Chinese News Headlines about Trending Events: A Complex Network Perspective

**DOI:** 10.1371/journal.pone.0122174

**Published:** 2015-03-25

**Authors:** Huajiao Li, Wei Fang, Haizhong An, Xuan Huang

**Affiliations:** 1 School of Humanities and Economic Management, China University of Geosciences, Beijing, China; 2 Key Laboratory of Carrying Capacity Assessment for Resource and Environment, Ministry of Land and Resources, Beijing, China; 3 Department of Energy and Mineral Engineering in the College of Earth and Mineral Sciences, The Pennsylvania State University, State College, Pennsylvania, United States of America; 4 Lab of Resources and Environmental Management, China University of Geosciences, Beijing, China; Hangzhou Normal University, CHINA

## Abstract

Because the volume of information available online is growing at breakneck speed, keeping up with meaning and information communicated by the media and netizens is a new challenge both for scholars and for companies who must address public relations crises. Most current theories and tools are directed at identifying one website or one piece of online news and do not attempt to develop a rapid understanding of all websites and all news covering one topic. This paper represents an effort to integrate statistics, word segmentation, complex networks and visualization to analyze headlines’ keywords and words relationships in online Chinese news using two samples: the 2011 Bohai Bay oil spill and the 2010 Gulf of Mexico oil spill. We gathered all the news headlines concerning the two trending events in the search results from Baidu, the most popular Chinese search engine. We used Simple Chinese Word Segmentation to segment all the headlines into words and then took words as nodes and considered adjacent relations as edges to construct word networks both using the whole sample and at the monthly level. Finally, we develop an integrated mechanism to analyze the features of words’ networks based on news headlines that can account for all the keywords in the news about a particular event and therefore track the evolution of news deeply and rapidly.

## Introduction

With the development and popularization of information and network technology, the Internet has become the main medium from which people obtain information and news. Helping solve a serious information overload problem [1], search engines are recognized as one of the most useful and popular services on the web [2, 3]. Generally, the web (and a search engine) is the first source a person turns to for information or news [4]. People have grown accustomed to inputting a few keywords into search engines and then clicking on one or more headlines out of the voluminous search results. Users can choose based on the closeness of the match and the users’ desire to obtain a detailed description of the news, and some scholars have successfully researched recommendation algorithms regarding the news and social networks based on users’ behavior and their similarities [5, 6, 7, 8]. Furthermore, more and more people realize that online news plays an important role in the spread of public opinion; thus, it is of great importance to know what and how different news sources present information. A headline is a significant component of the news and not only presents or relates the main points of news content but also must attract and hold the reader's attention [9]. Some scholars have provided evidence that there are connections among public relations, public awareness and news [10]. As networks develop, crisis communication theories require further modification and perfecting [11].

To analyze the information contained in news headlines, we should begin with information extraction technology. Information extraction can be traced back to 1960, when scholars first attempted to extract structured information from natural language text. In the news field, previous studies have mainly focused on text mining techniques and tools [12, 13], semantic analysis [14], analysis of sentiment [15, 16], etc. Some scholars have observed that news has value to an extent. Yoon (2012) observed that it is useful to detect weak signals for long-term business opportunities using text mining of web news [17]. Huang, Liao, Yang, & Chang (2012) proposed a financial news headline agent to assist with investment decisions in the Taiwanese stock market after receiving essential real-time news headlines disseminated by the agent [18]. Regarding text mining methods, scholars have studied data pre-processing [19], text mining [20, 21, 22] and visualization [23, 24]. Chen and Hsieh (2006) observed web page classification based on a support vector machine using a weighted vote schema [25]. Magerman, Van and Song (2010) explored the feasibility and accuracy of latent semantic analysis performed by text mining techniques and detected similarities between patent documents and scientific publications [26]. However, the majority of current theories and tools are directed at identifying one website or one piece of online news, although the information from one website or one piece of online news may be biased and insufficient to rapidly develop an understanding of a topic. As the trending event, there are hundreds of pieces of news reporting it, and there are thousands of words included in the headlines, the words can be linked to each other to form a big words network. Complex network theory can provide an improved approach to analyzing the evolution of the words network of news headlines.

Complex network theory has attracted great interest with respect to solving complex issues in recent years. A theory derived from the study of physics, complex network theory has been applied to many empirical studies, particularly in management [27], sociology [28, 29], and economics [30, 31, 32]. The fundamental principle of complex network theory is to identify units and relations between the units, which enables the construction of a network utilizing the units as nodes and the relations as edges to analyze and solve problems holistically.

In this paper, we chose the Deepwater Horizon oil spill (http://en.wikipedia.org/wiki/Deepwater_Horizon_oil_spill, which is also known as the 2010 Gulf of Mexico oil spill) and the 2011 Bohai Bay oil spill (http://en.wikipedia.org/wiki/2011_Bohai_Bay_oil_spill) as our empirical subjects or themes, and we gathered and pretreated all the Chinese news headlines in the search results on the two trending events from Baidu (http://www.baidu.com), the most popular and well-known Chinese search engine. We used Simple Chinese Word Segmentation to segment all the headlines into words and then used the words as nodes and the adjacent relations as edges to construct the words networks across the whole sample as well as at the monthly level. We integrated statistics, word segmentation, complex network and visualization to analyze all the headlines’ keywords and the evolution of online news about the two different trending events.

## Main Process and Data

### Main process

**Fig 1:**
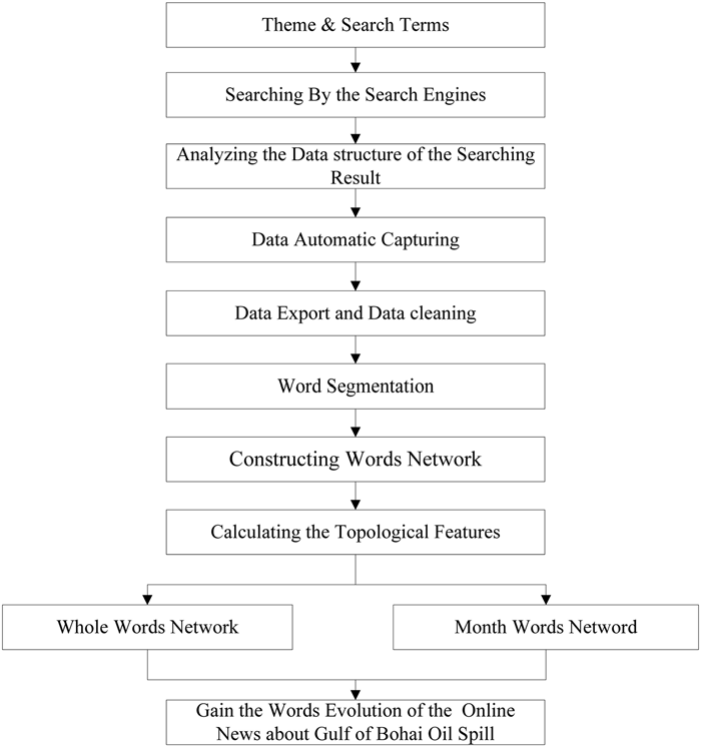
The main process of the research.

As shown in Fig. 1, the main process involved in this research occurs over nine steps. First, we chose the theme of the analysis and determined the search terms we would use. For this paper, as described above, we chose two trending events involving oil spills, the Gulf of Mexico oil spill, which occurred in May, 2010, and the Bohai Bay oil spill, which occurred in June, 2011. Second, we chose the search engines and input search terms to obtain the search results. In the third step, we analyzed and came to understand the rule of the search results and obtained the data structure to provide the foundation for Step 4. In Step 4, we developed tools to capture the search results automatically by inputting the data structure, which allowed us to export and clean the data based on the research object. In Step 5, we chose a suitable word segmentation tool to divide the Chinese headlines into different words. Steps 2 to 5 will be explained in more detail in section 2.2 below. After obtaining the words, we can construct different words networks according to the complex network methodology and calculate different features and analyze all the headlines’ keywords and the evolution of online news about the given theme. Steps 6 to Step 9 will be explained in detail in the remaining chapters of this paper.

### Data

The data used in this paper are mainly extracted from the Baidu (http://www.baidu.com) search engine, which is generally acknowledged as the most widely used search engine in China. We obtained the URL links for news regarding the two trending events from Baidu:

2010 Mexico oil spill: http://news.baidu.com/ns?word=%E5%A2%A8%E8%A5%BF%E5%93%A5%E6%B9%BE%E6%BC%8F%E6%B2%B9&pn=[*]&cl=2&ct=1&tn=news&rn=20&ie=utf-8&bt=0&et=0

2011 Bohai Bay Oil Spill: http://news.baidu.com/ns?word=%E6%B8%A4%E6%B5%B7%E6%B9%BE%E6%BC%8F%E6%B2%B9&pn=[*]&cl=2&ct=1&tn=news&rn=20&ie=utf-8&bt=0&et=0)

The relationship of the URL links and the pages can be expressed as following:

[*]=2p−10p=1,2,3,4,```,N (1)

where p is the online page number of Baidu News about the trending events, and N is the total number of pages of search results about the given hot event. In this research, there are a total of 38 pages regarding the 2010 Gulf of Mexico oil spill and 37 pages regarding the 2011 Bohai Bay oil spill.

In the pages, we captured the title, media source, date and time by two different labels: “<h3 class = "c-title"> </h3>” and “<p class = "c-author"> </p>”, and we automatically gathered all 1,487 pieces of Chinese news on 29 October 2014 about the 2011 Bohai Bay oil spill and the 2010 Gulf of Mexico oil spill. In the initial gathered data, there were 49 pieces of duplicate news form the same media at the same time and four news duplicate pieces of news before the event occurred in the 748 news stories about the 2010 Gulf of Mexico oil spill and 29 pieces of duplicate news from the same media at the same time and eight duplicate news pieces from before the event occurred in the 739 news about the 2011 Bohai Bay oil spill. Thus, after data cleaning, we obtained 695 pieces of news about the 2010 Gulf of Mexico oil spill and 702 pieces of news about the 2011 Bohai Bay oil spill.

**Fig 2:**
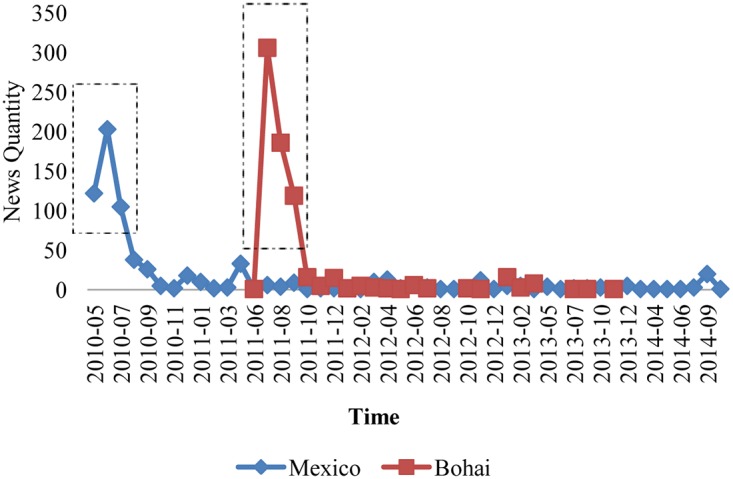
The evolution of the quantity of news after the events occurred.

Fig. 2 shows the time distribution of the news regarding the two trending events and enables us to find that both trending events were well covered in the media in the three or four months after each occurred, and then faded away to be talked about in the media only occasionally thereafter. Meanwhile, there is one notable difference between the news about the two trending events: the media first reported the 2010 Gulf of Mexico oil spill accident immediately after it occurred but first reported the 2011 Bohai Bay oil spill one month after it had occurred.

**Fig 3:**
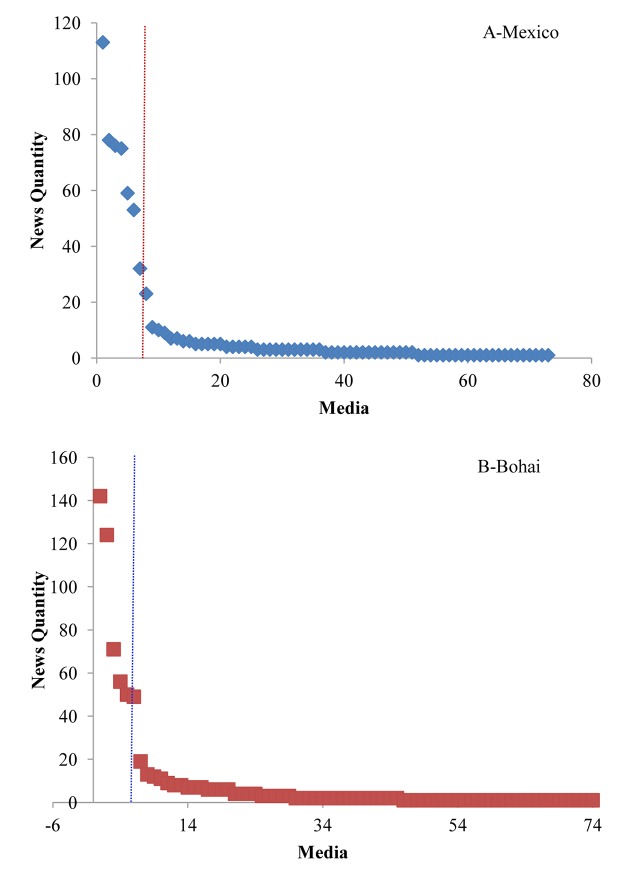
News Media Distribution of the two trending events.

Fig. 3 shows the media distribution of the two trending events. According to Fig. 3, only a few media outlets contributed the majority quantity of the news, and most media outlets reported no more than 10 pieces of news. The top six media outlets that reported the 2010 Gulf of Mexico oil spill are Netease, Sina, Xinhuanet, Sohu, Ifeng, Tencent, and the top six media that reported the 2011 Bohai Bay oil spill are Sina, Ifeng, Sohu, Hexun, Tencent, and Netease, which comprise all the mainstream media outlets in China.

## Method

### Method of headlines’ word segmentation

We used the open source word segmentation software called Simple Chinese Word Segmentation (http://www.xunsearch.com) based on the scripting language PHP. Simple Chinese Word Segmentation employs a dictionary containing more than 260 thousand Chinese words. The part-of-speech tagging used in this software is Peking University annotation, which contains 47 parts of speech. The input information is the headlines and the serial numbers of the headlines, whereas the output information consists of the serial numbers of the words, the words, the words’ part of speech, and the serial numbers of the headlines.

### Method of constructing words network

As described above, the main job of constructing the word network is to determine the nodes and edges as well as the weights of the edges. There are different ways of constructing networks, such as equivalence relationships (complete graph) [30], affiliation relationships (bipartite graph) [33, 42], and so on. In this paper, in order to show the words contextual relationships in the title, we gleaned the segmented words from the news headlines according to the features of the study subject (theme), and then we took each word as a node and connected nodes with edges based on the sequence of the words in the headlines, i.e., the former node as the start node and the node following the former node as the end node. This process was conducted repeatedly and sequentially for all the words in the titles.

**Fig 4:**
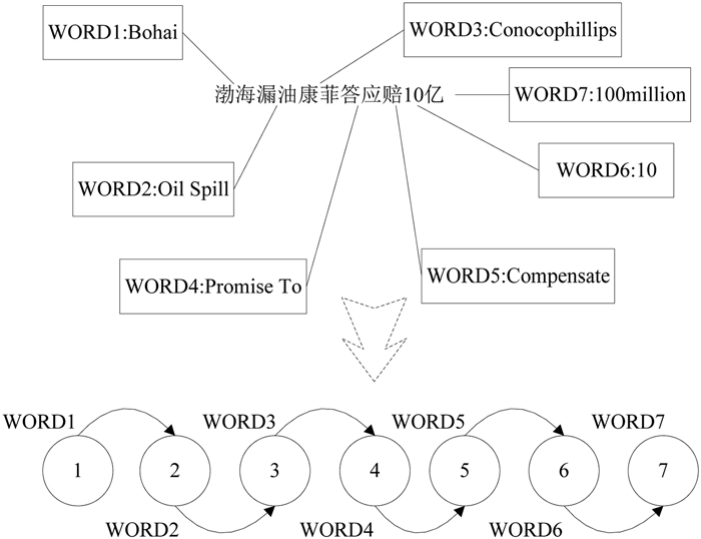
Construction of the Network (based on one title).

Fig. 4 shows the linear network for one title. Next, the linear networks of different headlines were superimposed; the weights of the edges are the times of the appearance of the edges between two nodes in different linear networks. Let graph G = (V,E,W) represent the directed weighted network in which V and E are the set of nodes and edges, and W represents the weight of the edges. Formula (2) shows the definition of the edges of the words in one title. In addition, the weight of the edge between two different nodes is the sum of $e_{ij}$.

$$\left\{ \begin{array}{c} {\text{e}}_{\text{ij}}\left(\text{k}\right)=1\;{\text{V}}_{\text{i}}{\text{is the nearby former node of V}}_{\text{j}}\text{in title k}\\ {\text{e}}_{\text{ij}}\left(\text{k}\right)=0\;{\text{V}}_{\text{i}}{\text{is not the nearby former node of V}}_{\text{j}}\text{in title k} \end{array}\right.$$ (2)

### The calculation methods of topological features

There are numerous topological features of the nodes, edges and networks in complex network theory. In this paper, we mainly analyzed the two different levels of networks using the following seven different topological features: degree, degree assortativity, weighted degree, average shortest path length, clustering coefficient, community structure, and stability coefficient. Meanwhile, on the basis of the distribution of the degree and the weighted degree of the words network, we analyzed the scale-free characteristics of the whole-sample words network, and we also analyzed the small-world properties of the words networks based on the average shortest path length and clustering coefficient between the words networks and the two different random networks, one with same average degree and another with same degree sequence as the words networks by network reshuffling [33].

The node’s degree indicates how many nodes connect. The more connections a node makes, the more importance that node has.

$R_{i}^{in}(t)$ represents the in-degree of node i:

$$R_{\text{i}}^{\text{in}}\left(\text{t}\right)=\Sigma_{\text{j=1}}^{\text{n}}{\text{e}}_{\text{ji}}$$ (3)

$R_{i}^{out}\left(t\right)$ represents the out-degree of node i:

$${ℛ}_{\text{i}}^{\text{out}}(\text{t})=\sum_{\text{j}=1}^{\text{n}}{\text{e}}_{\text{ij}}$$ (4)

$R_{i}\left(t\right)$ represents the sum of the in-degree and the out-degree of node i:

$$R_{\text{i}}\left(\text{t}\right)=R_{\text{i}}^{\text{in}}+R_{\text{i}}^{\text{out}}$$ (5)

In order to analyze the degree assortativity of the network, we use Pearson correlation coefficient of the degrees of any of the two nodes connected by a link to calculate it [34, 35].

$${\text{r}}^{\text{in}}=\langle r_{\text{i}}^{\text{in}}\rangle =\langle \frac{\left(R_{\text{i}}^{\text{in}}-\bar{R_{\text{i}}^{\text{in}}}\right)\text{*}\left(R_{\text{j}}^{\text{out}}-\bar{R_{\text{j}}^{\text{out}}}\right)}{\text{σ}\left(R_{\text{i}}^{\text{in}}\right)\text{σ}\left(R_{\text{j}}^{\text{out}}\right)}\rangle$$ (6)

$${\text{r}}^{\text{out}}=\langle r_{\text{i}}^{\text{out}}\rangle =\langle \frac{\left(R_{\text{i}}^{\text{out}}-\bar{R_{\text{i}}^{\text{out}}}\right)\text{*}\left(R_{\text{j}}^{\text{in}}-\bar{R_{\text{j}}^{\text{in}}}\right)}{\text{σ}\left(R_{\text{i}}^{\text{out}}\right)\text{σ}\left(R_{\text{j}}^{\text{in}}\right)}\rangle$$ (7)

where $r^{in}$ is the in-degree assortativity of the network, $r^{out}$ is the out-degree assortativity of the network, $\bar{R}$ is the average degree and  σ is the standard deviation of different degrees.

For a weighted network, the importance of a node is determined not only by the number of nodes it connects but also by the weight between the node and other nodes. The higher the weight, the more frequently the two words will appear together.

${WR}_{i}^{in}$ represents the in-weighted degree of node i:

$$\text{W}{ℛ}_{\text{i}}^{\text{in}}=\sum_{\text{j}=1}^{\text{n}}{\text{w}}_{\text{ji}}$$ (8)

$WR_{i}^{out}\left(t\right)$ represents the out-weighted degree of node i at time t:

$$\text{W}{ℛ}_{\text{i}}^{\text{out}}=\sum_{\text{j}=1}^{\text{n}}{\text{w}}_{\text{ji}}$$ (9)

${WR}_{i}\left(t\right)$ represents the sum of the in-weighted degree and the out-weighted degree of node i at time t:

$$\text{W}{ℛ}_{\text{i}}=\text{W}{ℛ}_{\text{i}}^{\text{in}}+\text{W}{ℛ}_{\text{i}}^{\text{out}}$$ (10)

Most real-world network distributions have long right tails of values that are far above the mean, and the degree distribution of the nodes obeys a power law according to M. E. J. Newman [36, 37]; thus, we say that the network has scale-free characteristics. In a scale-free network, the degree (weighted degree) distribution follows a power law:

$${\text{P}}_{\text{R}}\propto {\text{R}}^{-\lambda }$$ (11)

while λ can be calculated by:

$$\ln{\text{Ρ}}_{\text{R}}\propto -\text{λlnR}$$ (12)

The shortest path length of two words means the least quantity of edges between them. The average shortest path length represents the connectivity of different words as well as the words network, and it can be calculated by [38]:

$${\text{d}}_{\text{ij}}=\frac{1}{\text{N}\left(\text{N}-1\right)}\sum_{\text{i},\text{j}\in \text{N},\text{ i}\neq \text{j}}{\text{e}}_{\text{ij}}$$ (13)

The clustering coefficient means the connectivity of the neighbor nodes of a given node and is given by the ratio of existing edges (E_i_) between its first neighbors ($R_{i}$) to the potential number of such ties ($\frac{1}{2}R_{i}(R_{i}-1)$). In addition, we can obtain the clustering coefficient of the network by averaging the clustering coefficient of all nodes in the network. [39]

$$\text{C}=<{\text{C}}_{\text{i}}{>}_{\text{i}}=<\frac{2\text{Ei}}{{ℛ}_{\text{i}}\left({ℛ}_{\text{i}}-1\right)}>$$ (14)

Moreover, if the network presents a high probability that two neighbors of one given node are also connected themselves with a small average shortest path length between two nodes, we call the network a “small-world” network.

Meanwhile, the community structure and stability coefficient are two useful features that are often used in evolution analysis. In this paper, we used the heuristic method [40] and “auto-correlation function” (or “similarity coefficient function”) [41, 42] to analyze the evolution of the monthly-words networks. Formula (15) and Formula (16) shows the main step of the two different methods.

$$∆\text{M}=\left[\frac{\sum_{\text{in}}+{\text{k}}_{\text{i},\text{in}}}{2\text{m}}-{\left(\frac{\sum_{\text{tot}}+{\text{k}}_{\text{i}}}{2\text{m}}\right)}^{2}\right]-\left[\frac{\sum_{\text{in}}}{2\text{m}}-{\left(\frac{\sum_{\text{tot}}}{2\text{m}}\right)}^{2}-{\left(\frac{{\text{k}}_{\text{i}}}{2\text{m}}\right)}^{2}\right]\;$$ (15)

where $\sum_{in}$ represents the degrees of all the links inside the community K, $\sum_{tot}$ represents the total degrees of the nodes in K, $k_{i}$ represents the total degrees of node i, $k_{i,in}$ represents the total degrees of the links from i to all the nodes in K, and m represents the total degrees of the network. The community will be combined repeatedly until ∆M is negative while combining the communities.

$$\text{S}\left(\text{t}\right)=\frac{N_{\text{t}}\cap N_{\text{t}-1}}{N_{\text{t}}\cup N_{\text{t}-1}}$$ (16)

where $S\left(t\right)$ is the stability coefficient (similarity coefficient) of the words network. $N_{t}$ represents the set of nodes in the words network in month t, and $N_{t-1}$ represents the set of nodes in the network in month t-1. $N_{t}{\cap N}_{t-1}$ is the number of common nodes (words) at $N_{t-1}$ and $N_{t}$, and $N_{t}{\cup N}_{t-1}$ is the number of nodes at the union of $N_{t-1}$ and $N_{t}$.

## Results and Analysis

### The topological features of the whole-sample words network

#### The visualization of the whole-sample words network

**Fig 5:**
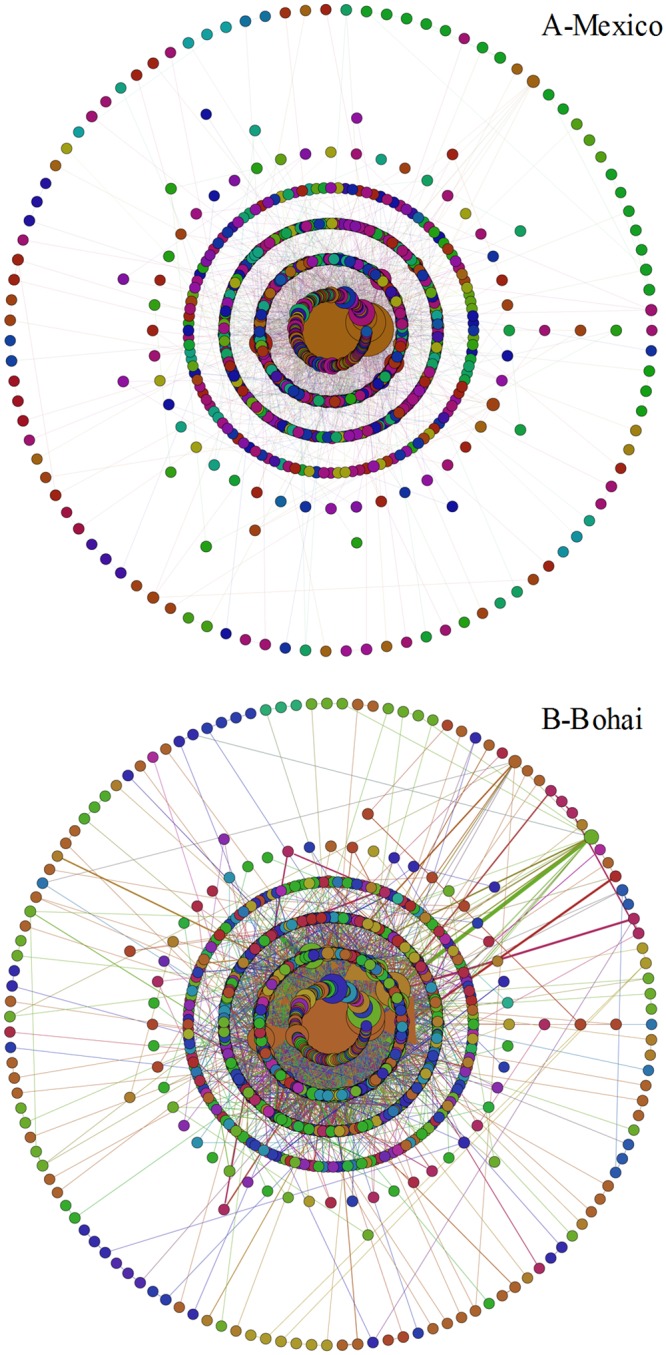
Visualization results of the whole-sample words networks of the two trending events.

After application of the Simple Chinese Word Segmentation software, we obtained 5,661 words regarding the 2010 Gulf of Mexico oil spill and 6,821 words regarding the 2011 Bohai Bay oil spill (after eliminating punctuations). After cleaning duplicate words, there were 1,288 different words in all the online Chinese news headlines regarding the 2010 Gulf of Mexico oil spill and 1,572 different words in all the online Chinese news headlines regarding the 2011 Bohai Bay oil spill, which means there are 1,288 nodes in the whole-sample words network about Mexico and 1,572 nodes in the whole-sample words network about Bohai. Fig. 5 presents the visualization results of the two whole-sample words networks regarding Mexico and Bohai (the color of the node is determined by the community Id which the node belongs to).

#### The scale-free characteristics and degree assortativity of the whole-sample words network

According to Formula (5) and Formula (10), we can calculate the degree and weighted degree of each node in the two whole-sample words networks and obtain the keywords of the two trending events in the whole-sample words network perspective. Tables 1 and 2 show the keywords of the two trending events (CNOOC represents “China National Offshore Oil Corporation”).

**Table 1:** Keywords of the 2010 Gulf of Mexico oil spill by degree and weighted degree.

Keywords By Degree		Keywords By Weighted Degree	
ID	Words	ID	Words
W00010	Oil Spill	W00010	Oil Spill
W00595	Gulf of Mexico	W00595	Gulf of Mexico
W00011	Event	W00011	Event
W00076	America	W00049	Oil
W00020	Accident	W00020	Accident
W00965	BP	W00077	Company
W00219	Already	W00076	America
W00049	Oil	W00023	100 Million
W00062	Probably	W00219	Already
W00012	Impact	W01001	Britain

**Table 2:** Keywords of the 2011 Bohai Bay oil spill by the degree and weighted degree.

Keywords By Degree		Keywords By Weighted Degree	
ID	Words	ID	Words
W00010	Oil Spill	W00010	Oil Spill
W00020	Accident	W00009	Bohai Bay
W00009	Bohai Bay	W00020	Accident
W00026	Conocophillips	W00069	CNOOC
W00011	Event	W00295	Bohai
W00025	Claims	W00026	Conocophillips
W00295	Bohai	W00011	Event
W00071	Declare	W00259	Oil Field
W00440	Pollution	W00071	Declare
W00069	CNOOC	W00025	Claims

**Fig 6:**
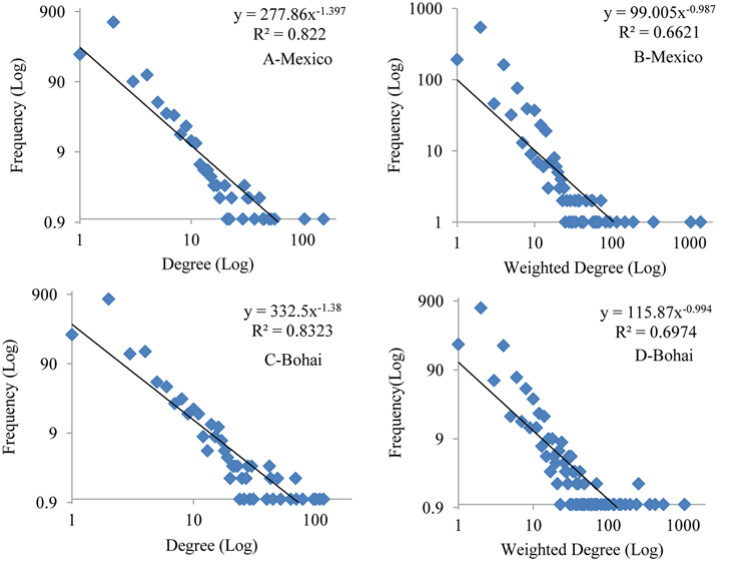
Degree and weighted degree distribution of the whole-sample words networks.

According to Fig. 6, both the degree distribution and the weighted degree distribution of the two whole-sample words networks can be approximated by the power-law $:\;{\ln Ρ}_{R}\propto -\lambda lnR,$, with good R^2^ (goodness of fit). Thus, we can conclude that the two networks are scale-free. Meanwhile, according to Formula (6) and Formula (7), we can get that the in-degree assortativity of two whole-sample words networks is 1.38671E-05 and -9.69644E-06, respectively, and the out-degree assortativity of two whole-sample words networks is-4.06877E-05 and -2.39787E-05, respectively, which are close to zero and much lower than the degree assortativity of real world networks [43]. So we can conclude that the words networks constructed in this paper have no significant assortative or disassortative mixing features.

#### The small world properties of the whole-sample words network

A small world network means that the neighbors of a given node have a high probability of contact with one another with a short average length. In the words networks, if it is small-world, it indicates that the words of the headlines contact very well with one another, and most of the points of the news are well connected. According to Formula (13) and Formula (14), we can gain both the average shortest path and the average clustering coefficient of the two whole-sample words networks. The average clustering coefficients of the two whole-sample words networks about the 2010 Gulf of Mexico oil spill and the 2011 Bohai Bay oil spill are 0.042 and 0.054, respectively. They are much larger than the clustering coefficients of the random networks of the identical size, which are both 0.001, as well as the random networks with the same degree sequence, which are 0.004 and 0.005, respectively. The average shortest path lengths of the two whole-sample words networks are 4.01 and 3.931. They are shorter than the random network with the same mean degree (1.969 and 2.27), which are 5.316 and 5.026, and the random network with the same degree sequence, which are 11.525 and 11.211. Thus, we can conclude that the two networks have small-world properties.

The results of scale-free characteristics and small world properties of the whole-sample words networks indicate that, the networks constructed in this paper are nonrandom and well- connected than the random networks with the same mean degree as well as the random networks with the same degree sequence as the words networks by network reshuffling. So, words in the online news titles are well connected by the regular grammatical rules and media preference of the words related to the topic of the trending events. However, by analyzing the degree assortativity, we can find that, most of them show very weak disassortative mixing, which is similar as model of Barab´asi and Albert and random networks [43].

### The evolution of the monthly-words network

#### The visualization of the monthly-words network

**Fig 7:**
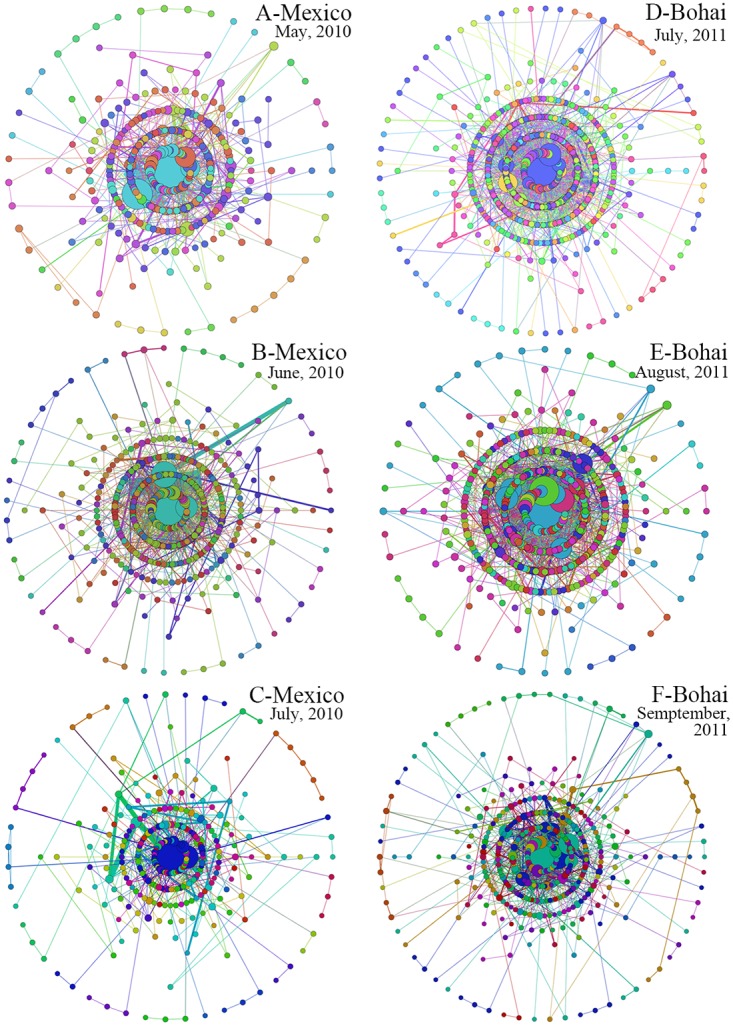
Visualization results of the monthly-words networks of the two trending events.

**Fig 8:**
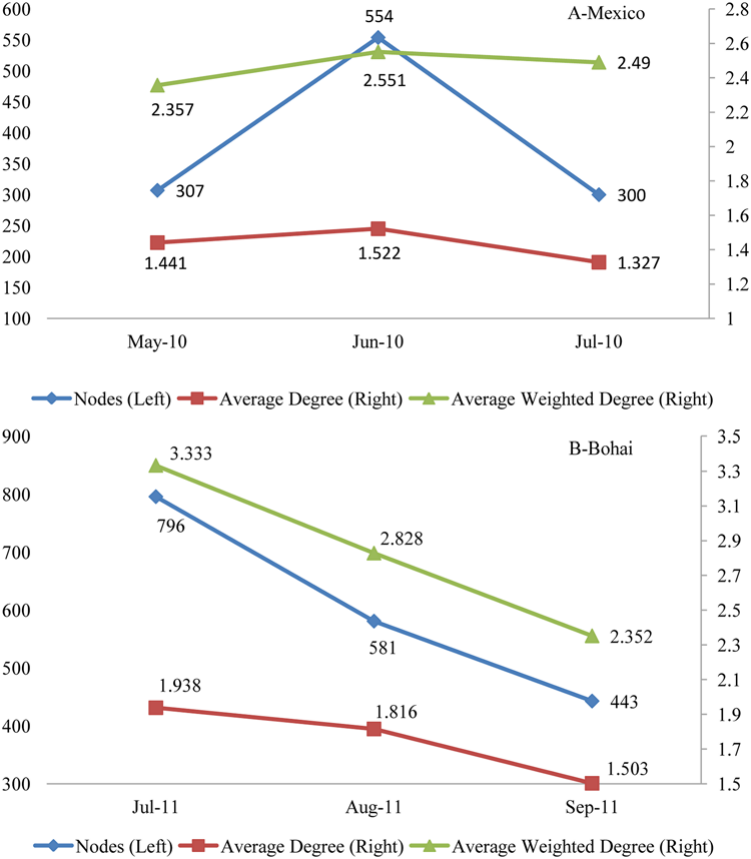
Evolution of nodes and the average degree and the average weighted degree of monthly-words networks about the two trending events.

To analyze the evolution of words in the headlines about the two trending events, we constructed the words networks for different months and analyzed the evolution of different topological features regarding the monthly-words networks. According to Fig. 2, the 2010 Gulf of Mexico oil spill was widely covered by the Chinese media immediately after it occurred, whereas the 2011 Bohai Bay oil spill was not widely covered by the Chinese media until one month after it occurred. Both events were hotly debated for approximately three months in the media; for the Gulf of Mexico oil spill, that time period was May-July 2010, whereas for the Bohai Bay oil spill, that time period was July-September 2011. Thus, in this paper, we construct three different monthly-words networks for each event and analyze their evolution. Fig. 7 shows the visualization results of the monthly-words networks of the two events (the color of the node is determined by the community Id which the node belongs to.), whereas Fig. 8 shows the evolution of nodes and the average degree and the weighted degree of monthly-words networks.

In Fig. 8, the circles represents the different distances between the words and the core keywords, we can discover that the most of the keywords (the big nodes) in different period are closely connected in the networks. Fig. 8 shows that both of the two trending events were most highly concerned by the media in the next month after they occurred. However, since 2010 Mexico oil spill were reported immediately by the parties responsible for the accident, and 2011 Bohai Bay Oil Spill were reported delayed by the the parties responsible for the accident, the online news about the 2010 Mexico oil spill were well published in the month it occurred, and reached the top one month later, then it declined slightly in the third month. Meanwhile, most of the media reported the 2011 Bohai Bay Oil Spill one month later after it occurred, and then it declined slightly in the next two months.

#### The keywords evolution of the monthly-words network

According to Formula (5) and Formula (10), we obtained the degree and weighted degree of each node in the six different monthly-words networks of the two trending events. Table 3 and Table 4 show the evolution of the Top 10 keywords regarding the two trending events when they were well covered by the Chinese media (D represents “Degree”, and WD represents “Weighted Degree”). According to the two tables, it is clear that both the trending events have 21 different keywords and that each of the trending events lasted three months. Comparing the Top 10 keywords between the two trending events reveals that the keywords similarity coefficient (Formula (16)) between the two events is only 20%, which means that most of the keywords of the two trending events are different. For a single hot event, there are clear features of evolution; for the 2010 Gulf of Mexico oil spill more of the media outlets were concerned with topics such as “disaster” and “control” as time went by, whereas for the 2011 Bohai Bay oil spill, the media became increasingly concerned with “claims” and “compensation” as time passed. Meanwhile, in the beginning, the media focused more on “CNNOC”, and later, more media attention focused on “Conocophillips”.

**Table 3:** Keywords evolution of the 2010 Gulf of Mexico oil spill.

Keywords		May, 2010		June, 2010		July, 2010	
		D	WD	D	WD	D	WD
W00010	Oil Spill	1	1	1	1	1	1
W00595	Gulf of Mexico	2	2	2	2	2	2
W00076	America	3	5	4	5		
W00011	Event	4	3	3	3	3	7
W00077	Company	5	7			6	4
W00020	Accident	6	4	8	4	8	
W00071	Declare	7					
W00012	Impact	8	10	9	9		
W00062	Probably	9		5	7		
W00013	Severe	10					
W00049	Oil		6	10	8	4	3
W00177	Quantity		8				
W00121	Over		9				
W00873	Disaster			6	6	7	
W00219	Already			7		5	10
W00682	sustain				10		
W01410	Success					9	5
W00263	Control					10	
W01895	Sealed						6
W00541	Announce						8
W01001	Britain						9

**Table 4:** Keywords evolution of the 2011 Bohai Bay oil spill.

Keywords		July, 2011		August, 2011		September, 2011	
		D	WD	D	WD	D	WD
W00010	Oil Spill	1	1	1	1	1	1
W00020	Accident	2	2	7	4	7	5
W00009	Bohai Bay	3	3	5	3	2	2
W00011	Event	4	6				
W00069	CNOOC	5	4	8			
W00295	Bohai	6	5	6	5		6(1)
W00440	Pollution	7	10(1)				
W00071	Declare	8	8	4	7	4	
W00203	Conceal	9	9				
W00219	Already	10	10(2)				
W00259	Oil Field		7			10	6(2)
W00026	Conocophillips			2	2	3	3
W00025	Claims			3	6		
W00077	Company			9(1)	8	5(2)	8
W00110	Compensation			9(2)		8	
W00012	Impact			9(3)			
W00023	100 Million				9		
W00187	Apologize				10		
W00053	Fund					5(1)	4
W00043	Still Not					9	10
W00315	To Set Up						9

Note: 100 million is a Chinese unit of quantity, Yi

#### The community evolution of the monthly-words network

**Fig 9:**
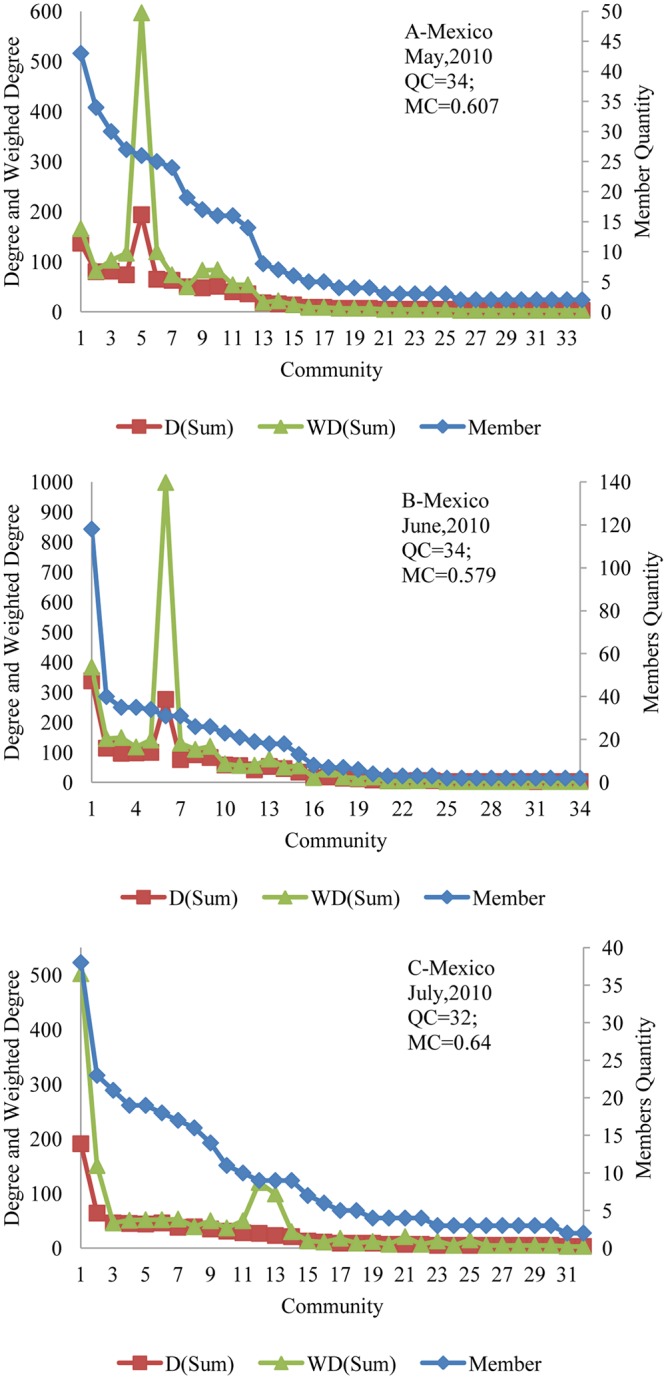
Evolution of monthly-words network community regarding the 2010 Gulf of Mexico oil spill.

**Fig 10:**
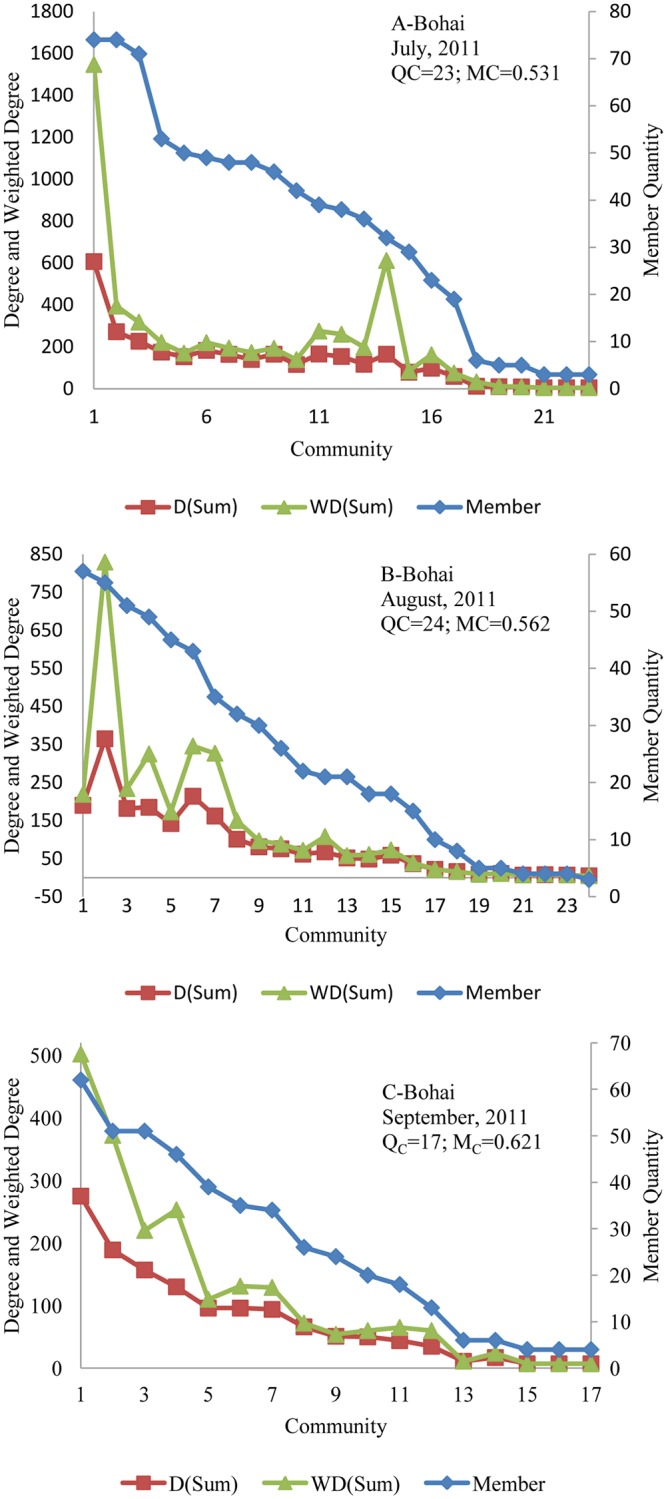
Evolution of monthly-words network community about the 2011 Bohai Bay oil spill.

According to Formula (15), the modularity (MC) means the independence between the communities, and the members in one community indicate that they have strong connections between one another. For the words networks, the nodes (words) in the same community indicate that they are well connected and more frequently appearing in same titles of the online news about the trending event. Fig. 9 and Fig. 10 show the members’ quantity and the total degrees and weighted degrees of each community in different monthly-words networks. Meanwhile, the two figures also show the evolution of the community quantity and modularity of each monthly-words network.

Clearly, in monthly-words networks, the community with the most members does not have the highest total degrees and weighted degrees. For example, in Fig. 9(a), the community with the most members is community 1, but the community with the highest total degrees and weighted degrees is community 5; as we analyzed the members of communities in detail, we found that community 5 contains five of the Top 10 keywords as its members, i.e., W00010, W00595, W00011, W00020, and W00071. Fig. 9 demonstrates that the modularity (MC) decreased from 0.607 to 0.579 and then increased from 0.579 to 0.64, which means the independence of the communities became weaker from May, 2010 to June, 2010, and then, it became stronger from June, 2010 to July, 2010. By contrast, the modularity (MC) of monthly-words networks regarding the 2011 Bohai Bay oil spill increased continuously from July, 2011 to September, 2011 according to Fig. 10.

**Fig 11:**
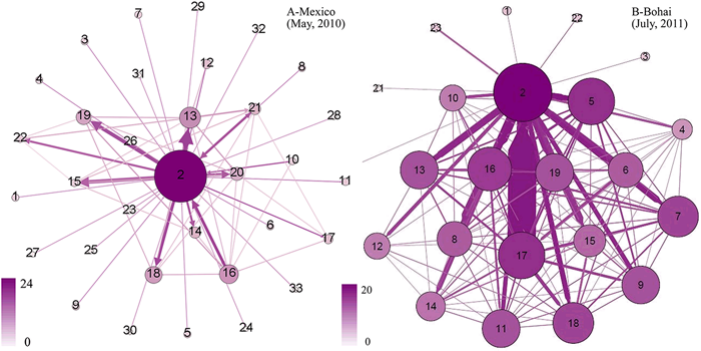
Community networks about the two trending events.

By comparison of Fig. 9 and Fig. 10, we can find that, although the nodes in monthly-words networks about the 2011 Bohai Bay oil spill are larger than the 2010 Gulf of Mexico oil spill, the community quantity of monthly-words networks about the 2011 Bohai Bay oil spill is much smaller than the 2010 Gulf of Mexico oil spill, which means that the words about the 2011 Bohai Bay oil spill are more focused on a few topics and that the words have stronger connections among one another. Fig. 11 shows the links between the communities of the two monthly-words networks, the 2010 Gulf of Mexico oil spill in May, 2010 and the 2011 Bohai Bay oil spill in July, 2011 (the color and size of the node is determined by the out-degree of community). It is obvious that, the communities of the monthly-words network about the 2010 Gulf of Mexico oil spill in May, 2010 is less linked than the communities of the monthly-words network about the 2011 Bohai Bay oil spill in July, 2011. It provides further evidence about why the modularity (MC) of the former monthly-words network is larger than the later one.

#### The evolution stability of the monthly-words network

**Fig 12:**
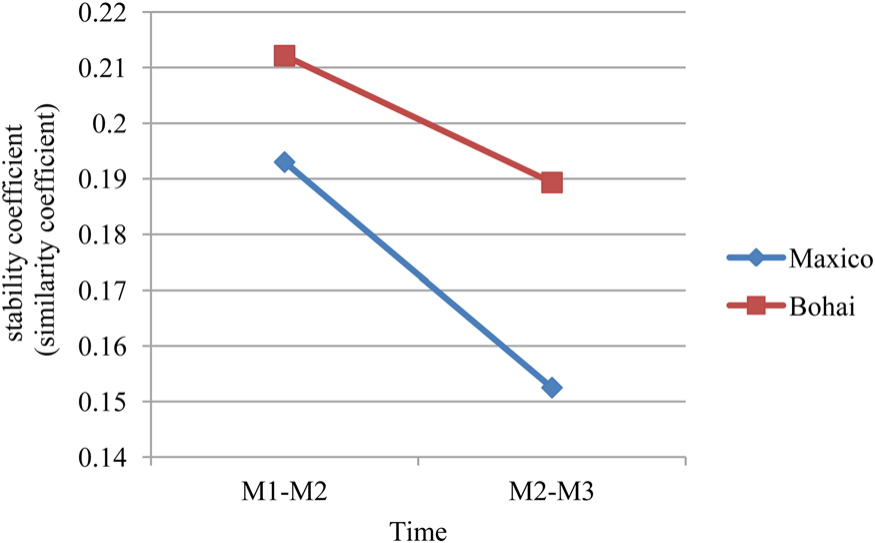
Stability coefficient of the monthly-words networks.

To analyze the stability and similarity of the words in different monthly-words networks, we used Formula (16) to calculate the stability coefficient of the monthly-words networks. According to Fig. 12, we can find that the stability coefficient of both the two trending events decreased gradually. In addition, the stability coefficients of the monthly-words networks regarding the 2011 Bohai Bay oil spill are larger than for the 2010 Gulf of Mexico oil spill, which means that the words regarding the 2011 Bohai Bay oil spill in different months are more similar. However, all four stability coefficients are less than 0.22, which indicates that most words appearing in the news headlines regarding the two trending events are new.

## Discussion and Conclusion

Complex network method has been well used in different empirical areas [44–48]. In this paper, we studied an infrequently considered but quite important method for developing a rapid and deep understanding of all the websites and all the news regarding one topic which integrates statistics, word segmentation, complex network theory and visualization to analyze all the online news headlines’ keywords and their evolution regarding two trending events, the 2010 Gulf of Mexico oil spill and the 2011 Bohai Bay oil spill.

We presented an integrated method to analyze both the whole-sample words network and monthly-words network regarding the online news headlines of the two trending events. Through our research, we found that, as with most empirical complex networks, the words networks of online news headlines regarding the two trending events have scale-free characteristics and small-world properties, and the degree assortativity coefficients of the two whole-sample words networks are very low. By calculating the topological features of the nodes, we obtain both the keywords of the whole-sample words network and the keywords of the monthly-words network. Meanwhile, we also obtained the inner relationship and evolution of the words. Compared with the 2010 Gulf of Mexico oil spill, we found that the words regarding the 2011 Bohai Bay oil spill are more focused on a few topics, and the connections between the words as well as the communities are stronger. We also found that both the words in the online news headlines regarding the 2010 Gulf of Mexico oil spill and the 2011 Bohai Bay oil spill changed obviously as time passed.

Such word-network analysis is a helpful tool with which scholars and companies may analyze and address the public concern regarding an event in a given theme. However, many problems remain to be studied. For example, some of the online news cannot be indexed by existing search engines. If we want to gather information regarding word networks more precisely, we must explore more methods to search the news. Therefore, in the future, we could extend the methods of data searching and try to construct the word networks of the headlines according to reality. Certainly, some of the titles are sensationalized or misleading, which does not reflect the real meaning of the contents of the news; thus, as a next step, we can identify a new method to judge the degree of correlation between the titles and the contents of the news.
